# Versatile and comprehensive hyperspectral imaging tool for molecular neuronavigation: a case study on cerebral gliomas

**DOI:** 10.1117/1.JBO.30.12.126007

**Published:** 2025-12-15

**Authors:** Dorotea Nardini, Anam Toaha, Camilla Bonaudo, Ivan Ezhov, Angelos Artemiou, Manuel Camelia, Filippo Nozzoli, Luca Giannoni, Ilias Tachtsidis, Alessandro Della Puppa, Daniel Rueckert, Pietro Ricci, Francesco Pavone

**Affiliations:** a University of Florence, Department of Physics and Astronomy, Sesto Fiorentino, Italy; b European Laboratory for Non-linear Spectroscopy, Sesto Fiorentino, Italy; c University of Florence, Azienda Ospedaliero-Universitaria Careggi, Neurosurgery, Department of Neuroscience, Psychology, Pharmacology and Child Health, Florence, Italy; d Technischen Universität München, Klinikum rechts der Isar, Munich, Germany; e University College London, Department of Medical Physics and Biomedical Engineering, London, United Kingdom; f Careggi University Hospital, Histopathology and Molecular Diagnostics, Florence, Italy; g Imperial College London, Department of Computing, London, United Kingdom; h Munich Center for Machine Learning (MCML), Munich, Germany; i National Institute of Optics, National Research Council, Florence, Italy

**Keywords:** hyperspectral imaging, glioma grading, spectral unmixing, contrast maps

## Abstract

**Significance:**

Accurate and timely characterization of brain tumors remains a major challenge in neurosurgery. Current intraoperative guidance relies on preoperative imaging modalities such as magnetic resonance imaging, positron emission tomography, or computed tomography, which are essential for surgical planning but become less reliable during surgery due to brain shift. Furthermore, postoperative tumor classification depends on histopathology, which requires weeks and can delay treatment decisions. No existing tool offers real-time, label-free, and spatially resolved biomolecular information to support both intraoperative guidance and early tissue assessment.

**Aim:**

We developed HyperProbe1.1 (HP1.1), a hyperspectral imaging system designed to acquire comprehensive molecular and metabolic information from brain tissue without the need for contrast agents or staining.

**Approach:**

HP1.1 captures reflectance images across a broad range of narrow spectral bands, enabling spatial mapping of hemoglobin, cytochrome c oxidase, and oxygen saturation. In addition, ultraviolet-excited autofluorescence imaging provides information on metabolic cofactors - nicotinamide adenine dinucleotide and flavin adenine dinucleotide - relevant for tumor characterization. The system was validated using standardized phantoms and *ex vivo* glioma samples.

**Results:**

HP1.1 demonstrated strong performance in detecting spectral features across phantoms and in distinguishing glioma tissues of different histological grades, enabling the generation of rapid and spatially resolved molecular contrast maps.

**Conclusions:**

By providing label-free, high-content, and rapid biomolecular imaging, HP1.1 represents a powerful platform for noninvasive tissue assessment in controlled experimental settings and paves the way for future intraoperative applications.

## 1 Introduction

Gliomas remain among the most challenging brain tumors to treat because of their infiltrative growth, high recurrence rate, and limited therapeutic options.^1^^,^^2^ Their clinical management relies on a complex workflow combining multiple diagnostic modalities throughout the preoperative, intraoperative, and postoperative stages. In the preoperative phase, well-established imaging techniques such as magnetic resonance imaging (MRI), computed tomography (CT), and positron emission tomography (PET) play a central role in surgical planning by providing structural and functional information on the lesion.^3^^–^^6^ However, their intraoperative utility is limited by spatial mismatches introduced during surgery due to brain shift^7^ and by the absence of real-time tissue-specific contrast capable of guiding resection decisions. To enhance intraoperative decision-making, fluorescence-guided surgery is commonly adopted, using contrast agents such as 5-aminolevulinic acid (5-ALA)^6^ or sodium fluorescein (SF)^8^^,^^9^ to improve visual discrimination between healthy and pathological tissue and to identify tumor margins.^10^ Fluorescein-assisted confocal laser endomicroscopy (CLE) further enables intraoperative cellular-resolution imaging of tumor architecture^11^ but relies on intravenous injection of SF, has a narrow field of view, and requires expert interpretation, which limits its broader adoption. These fluorescence-based methods, although clinically valuable, are constrained by pharmacokinetics, limited specificity across tumor types and grades, and the need for exogenous dyes that may not be suitable for all patients.

More recently, label-free optical techniques have been explored to provide rapid intraoperative tumor assessment.^12^ Lifetime-based fluorescence probes,^13^^,^^14^ which exploit the autofluorescence of endogenous tissue molecules, have shown potential for intraoperative tumor delineation, but their integration into standard surgical workflows remains challenging due to motion sensitivity, calibration requirements, and limited spatial coverage. Handheld Raman spectroscopy systems^15^^,^^16^ have also shown encouraging results for intraoperative detection of gliomas and meningiomas, enabling rapid identification of tumor tissue; however, most Raman approaches are point-based, measuring only small areas at a time and requiring mechanical scanning, which slows acquisition and limits coverage of tumor heterogeneity during surgery. As a result, comprehensive tissue characterization still largely depends on postoperative histopathological analysis, which remains the gold standard for tumor grading.^17^ This process, defined by the World Health Organization (WHO) 2021 classification,^18^ is essential for prognosis and treatment planning. Yet, it is inherently subjective,^19^ requires extensive sample preparation, and often introduces delays of weeks that may postpone critical decisions regarding adjuvant therapies. These limitations have motivated the development of faster, more objective tools to support intraoperative tissue assessment and early postoperative evaluation.

In this context, hyperspectral imaging (HSI) represents a promising noninvasive alternative for label-free characterization of brain tissue, with potential both to complement conventional histopathology and to support real-time decision-making during surgery.^20^ HSI captures both spatial and spectral information from tissue by acquiring a series of wide-field images at different, finely sampled wavelengths. These images are combined into a three-dimensional dataset—commonly referred to as a hyperspectral cube—in which each pixel contains a full reflectance spectrum across a broad and quasi-continuous spectral range. This spectral information reflects the attenuation properties of the tissue and can be used to discriminate between different tissue types or physiological states.^21^ However, despite this growing interest, the clinical adoption of HSI remains limited, partly due to technical challenges related to acquisition speed, system stability, spectral tunability, and signal-to-noise ratio—parameters that are critical for real-time and intraoperative use.

To address these limitations, we developed HyperProbe1.1 (HP1.1), a laboratory-based HSI system designed for the label-free analysis of brain tumor biopsies.^22^^,^^23^ Compared with our previous prototype (HP1.0),^24^ HP1.1 offers faster acquisition, a broader spectral range, higher spectral resolution, increased field of view (FOV), improved signal-to-noise performance, and enhanced tunability over single wavelengths—features that collectively support its applicability in surgical and early postoperative contexts.

The system was validated through a set of controlled laboratory experiments using synthetic optical phantoms and freshly excised human glioma biopsies obtained during neurosurgical procedures. Plastic optical phantoms with varying absorption and scattering coefficients were used to characterize spectral response,^25^^,^^26^ whereas fluorescent liquid phantoms allowed us to assess compatibility with conventional fluorescence-guided imaging protocols.^27^ Each measurement was followed by a streamlined spectral unmixing pipeline that estimates the relative abundance of relevant chromophores. This approach enabled early postoperative tumor grading, providing high-content molecular data without exogenous agents or extensive processing. It also generates real-time, spatially resolved contrast maps of vascularization and metabolism, potentially enhancing intraoperative neuronavigation and surgical decision-making.

Notably, HP1.1 also integrates an alternative imaging module based on selective light-emitting diode (LED) excitation and emission filtering, enabling the detection of autofluorescence signals from nicotinamide adenine dinucleotide (NADH) and flavin adenine dinucleotide (FAD), two metabolic cofactors recently validated as biomarkers for identifying and sorting glioma cancer cells.^28^^–^^30^ This added dimension strengthens the diagnostic capabilities of HP1.1 and highlights its potential as a comprehensive optical platform for neuro-oncological applications.

## 2 Materials and Methods

### 2.1 Optical Imaging Setup

The system employs two different illumination approaches: a broadband, wavelength-scanned illumination for hyperspectral reflectance imaging, and a monochromatic LED illumination for autofluorescence imaging.

For hyperspectral reflectance imaging, a broadband plasma light source (XWS-65, ISTEQ, spectral range 250 to 2500 nm, average output power via fiber 560 mW) is filtered by a tunable wavelength selector (FWS-Poly-CUS-10, Spectrolight). The selector performs hyperspectral scanning by sequentially transmitting narrow spectral bands (3 to 15 nm FWHM) between 385 and 1015 nm, with a switching time of ∼200  ms. The selected light is coupled into a 1-mm core optical fiber and delivered to the sample, providing an output power of about 1 mW at the sample plane, measured with a Thorlabs PM100D power meter.

For autofluorescence imaging, a single 370-nm LED (SST-08-UV-A130H-F365-00, Luminus) is used to simultaneously excite NADH and FAD, delivering ∼3  mW on the sample plane, measured under the same conditions as above.

The fiber output and the LED source were mounted on the wall of a custom 3D-printed plastic cone, specifically designed to minimize environmental light interference and to ensure precise alignment of the illumination at an angle of ∼35  deg with respect to the sample, thereby avoiding specular reflection. The reflected or emitted light was collected through an apochromatic macro-objective (SDF PLAPO 1X PF, Olympus) and relayed to the detection path. For autofluorescence acquisitions, the emitted signal was spectrally filtered using a motorized filter wheel equipped with two bandpass filters: a MV450-20 (450±10  nm) and a MV520/20 (520±10  nm) (Chroma Technology Corp.)—corresponding to the emission bands of NADH and FAD, respectively. The transmitted light was then focused by a tube lens onto a scientific CMOS camera (pco.panda 4.2, PCO), featuring a 2048×2048-pixel sensor, a maximum frame rate of 40 fps, and a quantum efficiency up to 80% in the visible–NIR range.

**Fig. 1:**
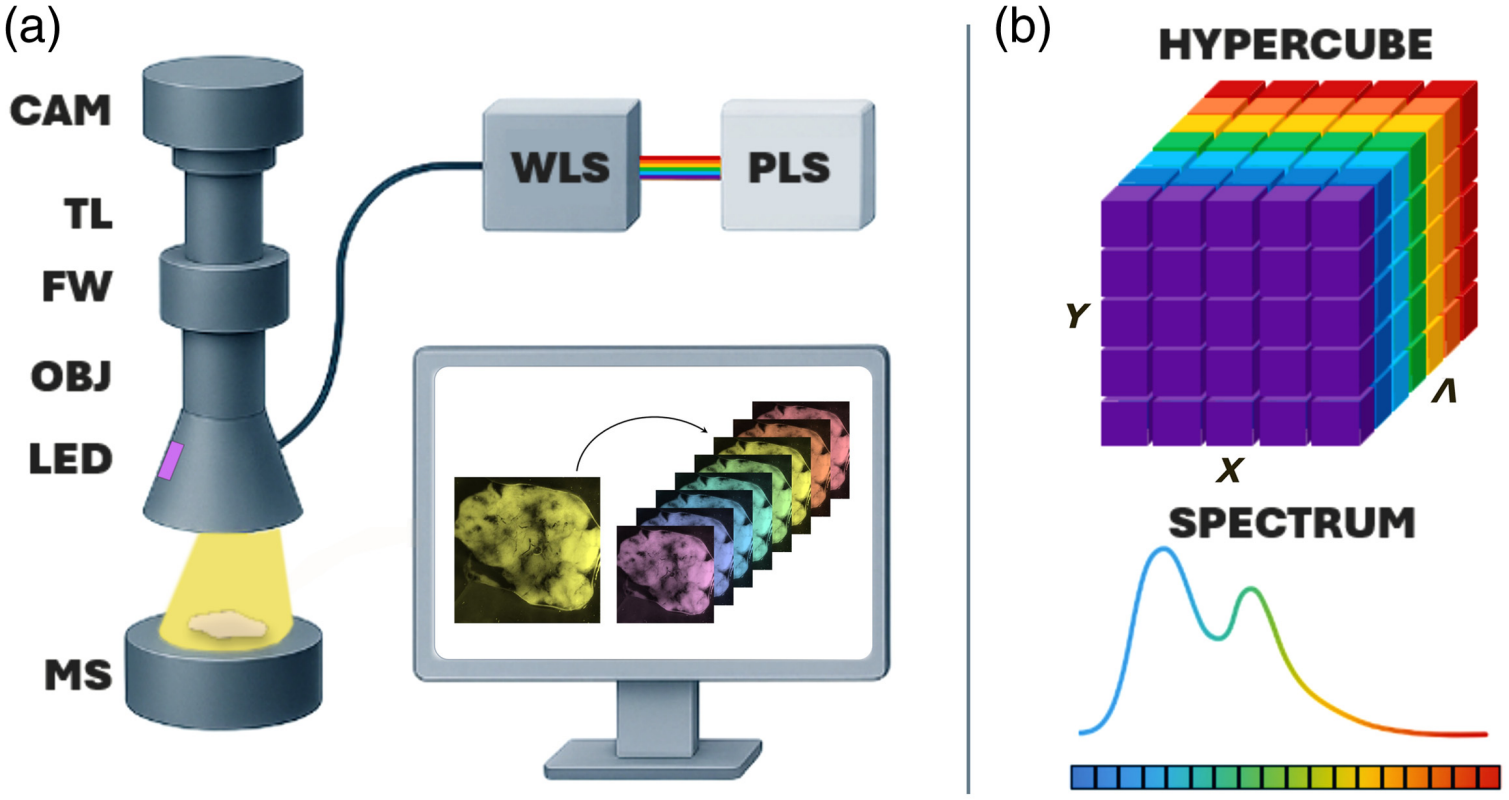
(a) Schematic representation of the HP1.1 and its components. In yellow, the light emitted from the optical fiber is shown as an example output, whereas sequential frames acquired at different wavelengths are depicted in multiple colors to illustrate the different illumination. PLS, plasma light source; WLS, wavelength selector; CAM, camera; TL, tube lens; FW, filter wheel; OBJ, objective; MS, motorized stage. (b) Hyperspectral cube generation. 2D reflectance images are sequentially acquired for each wavelength and stacked together. The resulting stack forms a hyperspectral cube with spatial (X,Y) and spectral (Λ) dimensions.

The physical arrangement of the system is shown in Fig. 1(a): the plasma light source, wavelength selector, optical assembly, and control computer are mounted on a compact wheeled optical rack, whereas the objective, filter wheel, tube lens, and CMOS camera are arranged vertically above the sample in a rigid, compact frame.

### 2.2 Hypercube Formation

The hyperspectral cube generation begins with a sequential illumination of the sample selectively filtering the broadband emission and projecting a single wavelength at a time. For the system characterization and validation, a spectral range of 385 to 1015 nm is set, with a spectral separation of 5 nm and a spectral bandwidth of 5 nm. For each selected wavelength, the system captures a two-dimensional grayscale reflectance image of the sample using a camera in widefield configuration. This process is repeated across multiple wavelengths, generating a stack of images—a three-dimensional hyperspectral cube—where the X and Y dimensions represent the spatial coordinates of the sample, and Λ encodes the spectral response at each pixel [Fig. 1(b)]. Notably, HP1.1 enables rapid acquisition of a complete hypercube in about 30 s per sample. This duration includes both the integration time for each frame (typically set at 50 ms) and the mechanical dwell time required by the wavelength selector to switch between each wavelength (∼200  ms).

### 2.3 Data Calibration and Processing

Periodically, the system was calibrated to compensate for spectral non-uniformities in both illumination and detection. The calibration procedure involved illuminating a white reference target (Labsphere, Spectralon^®^ 5″, nominal reflectance 99%) with all wavelengths emitted by the plasma light source, filtered by the wavelength selector, and capturing each reflectance image at a fixed integration time (e.g., 50 ms). This yielded the system’s response spectrum, reflecting the combined contributions of the emission spectrum from the wavelength selector, the chromatic response of the objective and tube lens, and the wavelength-dependent quantum efficiency of the CMOS sensor. Because the system does not allow direct control of excitation intensity, a time calibration was implemented to flatten and homogenize the system response. By setting a nominal integration time in the acquisition software, a corrective factor automatically adjusts the integration time for each wavelength, ensuring a uniform response across the spectrum.

For each sample, three hypercubes were acquired sequentially: a reference hypercube $I_{\text{white}}$, taken on the white plate; a dark hypercube $I_{\text{dark}}$, acquired with the plasma light source turned off; and the sample hypercube $I_{s}$. The reflectance hypercubes R(x,y,λ) for each sample were reconstructed by normalizing the hyperspectral sample data $I_{s}(x,y,\lambda )$, with $I_{\text{white}}(x,y,\lambda )$, after subtracting the dark counts $I_{\text{dark}}(x,y,\lambda )$ as in the following expression:

$$R(x,y,\lambda )=\frac{I_{s}(x,y,\lambda )-\frac{t_{s}}{t_{d}}\cdot I_{\text{dark}}(x,y,\lambda )}{\frac{t_{s}}{t_{w}}\cdot I_{\text{white}}(x,y,\lambda )-\frac{t_{s}}{t_{d}}\cdot I_{\text{dark}}(x,y,\lambda )},$$ (1)

where different nominal integration times $t_{s}$, $t_{d}$, and $t_{w}$ can be used for sample, dark, and white hypercubes acquisitions, respectively. This post-processing step ensures not only to remove background contributions but also to compensate for minor spatial illumination inhomogeneities.

Signal attenuation A(x,y,λ), comprehensive of the absorption and scattering of light with the sample, is directly obtained from the Beer-Lambert Law (BLL) as follows:

A(x,y,λ)=−Log R(x,y,λ). (2)

Finally, by periodically recalibrating the system and acquiring white and dark hypercubes for each measurement, we compensated for possible slow or periodic fluctuations in the illumination and detection paths, as well as for small temporal drifts in the light source. All measurements were performed with the laboratory lights switched off to prevent any interference from ambient illumination, ensuring accurate and reproducible reflectance data.

### 2.4 Spectral Unmixing

Building on our previous work,^31^ we employed an in-house implementation of a modified BLL-based spectral unmixing algorithm, publicly available for reproducibility.^32^ The method uses a standard nonlinear optimization solver adapted to the BLL formulation, typically requiring about 2 to 3 min to run on a dual AMD EPYC 7452 processor setup. This algorithm processes the optical signal at each image pixel to quantify the relative contributions of key biological endmembers. The analysis was performed in the 500 to 900 nm spectral window—a range selected for its optimal tissue penetration due to reduced light scattering and low water absorption. This range also encompasses the main absorption features of our target molecules: oxy-hemoglobin (${\mathrm{HbO}}_{2}$), deoxy-hemoglobin (HHb), lipids, water, and both oxidized (oxCCO) and reduced (redCCO) forms of cytochrome c oxidase, cytochrome b (Cyt_b), and cytochrome c (Cyt_c). To isolate molecular absorption signals while minimizing background contributions, the algorithm infers pixel-level concentration differences relative to a reference spectrum, defined as the average spectral reflectance from the central region of one representative sample in the dataset. This normalization effectively cancels constant or slowly varying spectral components across samples, enhancing sensitivity to local molecular variations.

For downstream analysis of malignancy grade separation, we derive the mean concentration of each molecular component per sample. These compositions, expressed as concentrations (μM/cm) or volumetric contents (${\mathrm{cm}}^{-1}$), are then jointly visualized to assess their discriminative potential across grades.

### 2.5 Samples for System Validation

A full set of plastic phantoms (MEDPHOT, BioPixS) was used to measure the performance of the HP1.1. These homogeneous plastic disks, characterized by varying absorption and scattering coefficients (see Table 1), provide a reliable standard for validating the system across a wide range of optical properties.^25^^,^^26^ Among them, the B3 disk is particularly relevant, as it mimics the optical properties of human brain tissue.

**Table 1:** Absorption and scattering coefficients of the MEDPHOT plastic disks at 690 nm, expressed in ${\mathrm{cm}}^{-1}$.

	A2	A3	A5	A7	B2	B3	B5	B7	C2	C3	C5	C7	D2	D3	D5	D7
Ab.	0.06	0.12	0.24	0.36	0.06	**0.12**	0.23	0.36	0.06	0.11	0.23	0.35	0.06	0.11	0.23	0.35
Sc.	5.9	6.2	6.1	6.2	11.0	**11.1**	11.3	11.8	16.5	16.0	16.6	16.3	21.0	21.3	21.8	22.4

To further evaluate the system’s capabilities, five different liquid phantoms were prepared. One phantom, used to assess HP1.1’s sensitivity, consisted of a 5-μM SF solution (260983T, VWR Chemicals) in water, tested at various dilutions. The remaining four phantoms were designed to demonstrate HP1.1’s ability to distinguish between standard intraoperative fluorescent dyes at different concentrations. For two of them, 10 mL of a 1.3% intralipid stock solution—prepared by diluting 2 mL of a 20% native intralipid (I141, Sigma-Aldrich) in 28 mL of deionized water—was mixed with 10 and 40 mL of the 5  μM SF solution, respectively. For the final two phantoms, 10 mL of the same 1.3% intralipid solution was mixed with 0.1 and 0.4 mL of 1.5 mM solution of protoporphyrin IX (PpIX; P8293, Sigma-Aldrich) prepared in dimethyl sulfoxide (DMSO; D4540, Sigma-Aldrich).

### 2.6 Glioma Sample Preparation and Histological Grading

A total of 31 patients with suspected glioma were enrolled in this study between December 2024 and July 2025 at the Azienda Ospedaliero-Universitaria Careggi (University Hospital of Florence). The study was approved by the Ethical Committee of the Area Vasta Centro Toscana (Study ID: 23672 - 23672_BIO), and informed consent was obtained from all participants, in accordance with Italian regulations. All surgical procedures were performed by the same senior neurosurgeon (A.D.P.), with the support of intraoperative neuronavigation (Stealth Medtronic) and neurophysiological monitoring. Tumor resections were performed during standard neurosurgical procedures, and fresh tissue samples were collected intraoperatively. Margin or healthy brain areas were not included for ethical reasons, as resection of normal brain tissue is not part of standard glioma surgery. Each specimen was divided into two adjacent portions: one was analyzed *ex vivo* using HP1.1, whereas the other underwent routine histopathological processing with hematoxylin and eosin (H&E) staining and IDH1 R132H immunohistochemistry^33^ and was classified according to the 2021 WHO grading system.^18^ Tumor grade and type were rigorously established through the histopathological diagnosis, which represents the clinical gold standard and provides a single categorical result for the entire specimen. Therefore, the two portions, both derived from the same surgical biopsy, shared the same pathological classification, which was consistently used as the ground truth reference for hyperspectral analysis.

Three samples were excluded due to inconclusive histopathological findings, resulting in a final dataset of 28 glioma cases (from 19 male and 9 female patients; mean age 55.5 years). All lesions were located supratentorially, with 12 in the right hemisphere, 14 in the left, one frontal bilateral, and one involving the corpus callosum; 9 lesions extended across more than one lobe. A dataset summary, comprehensive of the distribution of WHO grades, is provided in Table 2.

**Table 2:** Patients’ cohort and dataset information.

Samples	Grade 1	Grade 2	Grade 3	Grade 4	M:F	Mean age
28	1	5	3	19	19:9	55.5

Before imaging with HP1.1, the samples—ranging from 0.5 to 2 cm in size—were rinsed in phosphate-buffered saline (PBS) to remove blood and debris. A thin glass coverslip was placed on top to flatten the surface and ensure a uniform focal plane across the FOV, whereas a dark, light-absorbing substrate was used underneath to minimize reflections. Imaging was performed within 1 h of excision to preserve tissue integrity.

For large samples, multiple nonoverlapping acquisitions were performed, translating the FOV in distinct regions using a motorized stage. The displacement was programmed to exceed the FOV and was visually verified in real time through the live display to ensure complete spatial separation between consecutive acquisitions.

### 2.7 NADH and FAD Autofluorescence

To characterize the optical properties of NADH and FAD, standard synthetic solutions (N8129, Sigma-Aldrich, and F6625, Sigma-Aldrich) were first analyzed for their spectroscopic response. Absorption and emission spectra of both metabolic fluorophores were acquired using a 1-cm path-length quartz cuvette. A spectrophotometer (Lambda 950, Perkin Elmer) was employed to record absorption spectra, whereas a spectrofluorimeter (LS 55, Perkin Elmer) was used to acquire emission spectra under 370-nm excitation. In each case, the solvent spectrum (10 mM NaOH and H_2_O for NADH and FAD, respectively) was subtracted from the raw data.

To test the optical response of the autofluorescence imaging system and retrieve calibration coefficients, preliminary measurements were performed on two fluorescent liquid phantoms: a 281.9-μM NADH solution diluted in 10-mM NaOH and a 120.5-μM FAD solution diluted in H_2_O. Characterization measurements with HP1.1 were therefore performed on mixtures of NADH and FAD phantoms at different dilutions, as reported in Table 3.

**Table 3:** Dilutions and corresponding concentrations of the liquid phantoms used for preliminary NADH and FAD autofluorescence analysis.

NADH %	$x_{\mathrm{NADH}}$ (μM)	FAD %	$x_{\mathrm{FAD}}$ (μM)
0	0	100	120.5
20	56.4	80	96.4
40	112.8	60	84.4
50	141.0	50	60.3
60	169.1	40	48.2
80	225.5	20	24.1
100	281.9	0	0

Subsequently, 16 glioma samples of the tumor dataset were illuminated with a 370-nm LED source for autofluorescence imaging of NADH and FAD. Two images were acquired for each sample, using 450/20 and 520/20 nm emission filters placed in front of the camera, centered at the fluorescence peaks of the two molecules. The camera exposure time was set to 200 ms to maximize the collected signal while avoiding pixel saturation under both experimental conditions.

## 3 Results

### 3.1 System Characterization

In this study, we conducted a comprehensive characterization of HP1.1, evaluating image quality metrics such as spatial resolution and signal-to-noise ratio, along with spectral performance, including temporal stability, linearity, spectral range, and sensitivity.

**Fig. 2:**
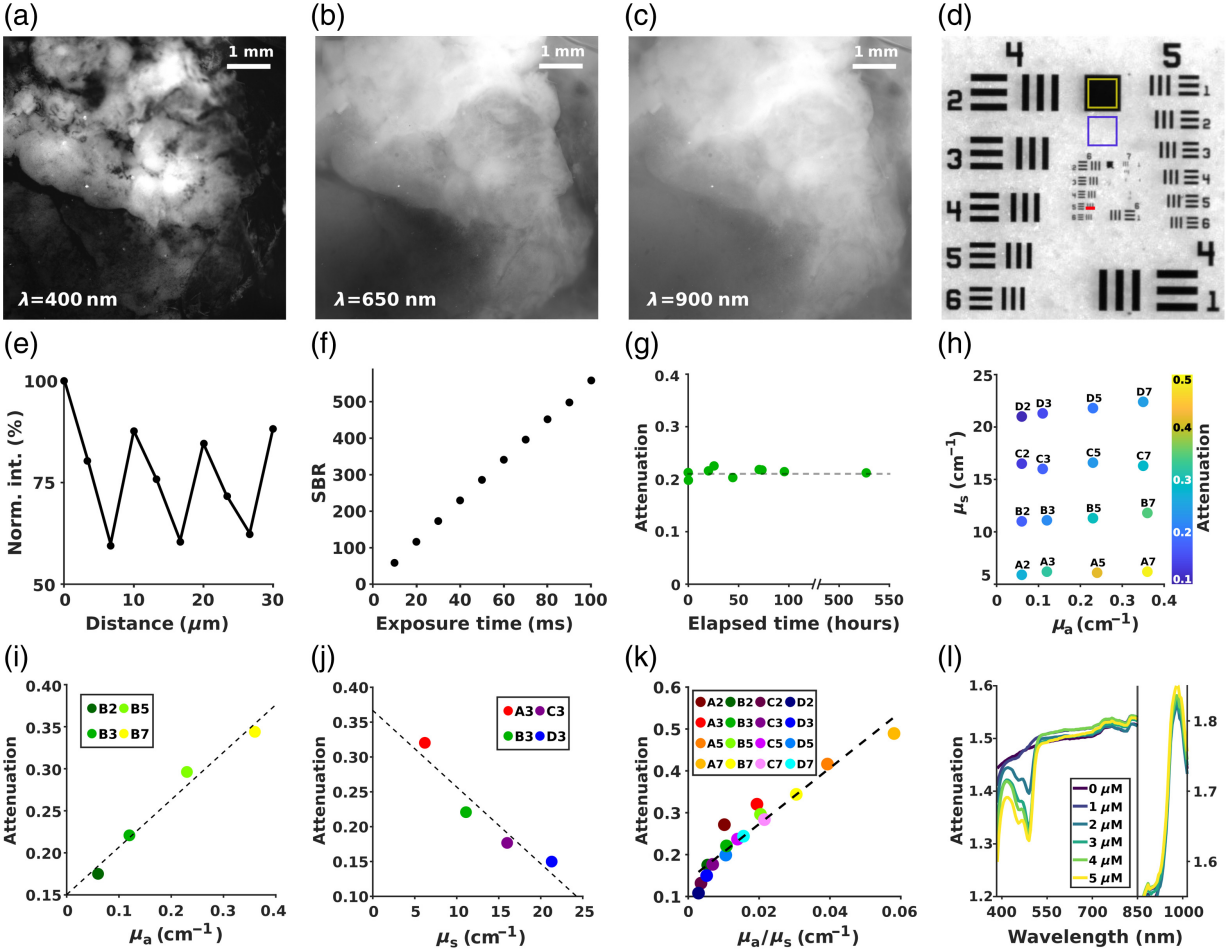
(a–c) High-grade glioma imaged with different wavelengths of illumination, 400, 650, and 900 nm, respectively. (d) USAF target imaged with 650 nm illumination light; yellow and purple regions are selected for contrast evaluation. (e) Normalized intensity profile over the red line shown in panel (d), corresponding to USAF’s group 6 element 5. (f) SBR trend against different exposure times, obtained from images with 650 nm illumination. (g) Attenuation signal from the disk B3 at 690 nm over several days. (h) Measured attenuation signal at 690 nm as a function of absorption and scattering coefficients of the plastic disks. (i–k) Linear relation between measured attenuation at 690 nm and the absorption and scattering coefficients and their ratio, respectively. (l) Attenuation spectra of different concentrations of fluorescein solution in water.

Figures 2(a)–2(c) show reflectance images of a cerebral glioma acquired at three representative wavelengths within the system’s spectral range (400, 650, and 900 nm), revealing different structures depending on the wavelength. This is a consequence of the light-tissue interaction, revealing molecule absorption and scattering dependency on the illumination wavelength. At 400 nm, contrast is high, and finer morphological details are visible, likely due to strong absorption by hemoglobin and related chromophores, which dominate in the blue-visible spectrum. At 650 nm, the contrast is moderately reduced, consistent with a lower absorption by oxygenated hemoglobin, allowing slightly deeper features to emerge. In the near infrared (900 nm), the reduction in both absorption and scattering permits greater light penetration, but the image becomes more diffuse and less defined, reflecting the trade-off between depth sensitivity and spatial resolution. These observations confirm that selecting specific spectral windows allows the system to target either morphological or biochemical information, depending on the surgical or diagnostic need.

To evaluate the contrast and spatial resolution of HP1.1, we acquired an image of a USAF resolution target under 650 nm illumination [Fig. 2(d)]. The target was placed on a neutral white background, and the resulting image displays sharp edge delineation of fine patterns and high contrast, highlighting the system’s optical performance. To quantify contrast performance, we measured the average intensity in two representative regions: a homogeneous white area (purple square, $I_{\max}$) and a low-reflectance black region (yellow square, $I_{\min}$). A contrast value of C=∼70% was computed as^34^

$$C=\frac{I_{\max}-I_{\min}}{I_{\max}+I_{\min}}$$ (3)

Indicating excellent dynamic separation between dark and bright areas, an essential feature to distinguish fine spatial features against complex backgrounds, especially when operating at short exposure times.

To assess spatial resolution, an intensity profile, shown in Fig. 2(e), was extracted across the red line drawn over the group 6 element 5 (line spacing: 4.92  μm). The profile reveals well-defined periodic peaks and valleys, confirming the system’s ability to resolve features at nearly cellular scale over a FOV of 6.5 mm. Therefore, HP1.1 supports both high-magnification imaging and wide-field acquisition when using lower magnification optics, making HP1.1 a flexible tool for intraoperative applications where both detail and coverage are needed.

To further validate the system performance as a function of exposure time, we measured the signal-to-background ratio (SBR) as a critical metric for real-time imaging scenarios. White reference images, taken as signal, were acquired under 650 nm illumination while varying the exposure time from 10 to 100 ms. For the same exposure settings, background images were taken without any reflecting target. The SBR, defined as the ratio between the mean signal calculated over the entire FOV in the white image, and the corresponding background image, is plotted in Fig. 2(f). A linear relationship was observed across the tested range, consistent with a system where signal intensity scales with illumination duration while background remains nearly constant. This suggests that background contributions are dominated by time-invariant sources, such as electronic noise. Exposure times beyond 100 ms were avoided to prevent sensor saturation; however, this constraint limited the SBR analysis to low exposure times, beyond which the SBR could show different, nonlinear trends. Nevertheless, the linearity observed in the tested interval confirms the predictable behavior of the system under the usual glioma acquisition conditions.

Spectral stability and spectral fidelity were evaluated using tissue-mimicking solid phantoms with known optical properties (see Table 2, Materials and Methods). Among them, we selected disk B3, which mimics biological tissue with an absorption coefficient $\mu_{a}=0.12\;{\mathrm{cm}}^{-1}$ and scattering coefficient $\mu_{s}=11.1\;{\mathrm{cm}}^{-1}$, imaging it repeatedly over hours, days, and weeks under consistent conditions at 690 nm. As shown in Fig. 2(g), the mean reflected intensity remained stable over time, indicating excellent temporal consistency of the system. The minor fluctuations observed may be attributed to small variations in the emission power of the light source—despite being measured as stable (∼1  mW) at the fiber output for each illumination wavelength. These fluctuations might also arise from environmental temperature shifts or slight misalignments during repositioning, although their impact was minimal, supporting the system’s reliability for longitudinal imaging and repeated measurements.

To explore the system’s response to materials with different optical properties, we measured signal attenuation across a set of calibration phantoms with varying absorption ($\mu_{a}$) and scattering ($\mu_{s}$) coefficients [Fig. 2(h)]. Linear trends were observed when attenuation was plotted independently versus $\mu_{a}$ [Fig. 2(i)] and $\mu_{s}$ [Fig. 2(j)]. Importantly, when the attenuation was plotted against the ratio $\mu_{a}/\mu_{s}$ [Fig. 2(k)], a strong linear correlation persisted, confirming that HP1.1 maintains quantitative response across diverse optical regimes. This behavior is crucial when interpreting spectra from heterogeneous tissues, where absorption and scattering vary concurrently and influence contrast mechanisms. The ability to reliably track these variations strengthens the system’s potential for performing spectral unmixing and quantification of biological signals.

Finally, we tested HP1.1’s sensitivity in detecting low concentrations of fluorescent contrast agents, imaging aqueous solutions of fluorescein at increasing concentrations from 0 to 5  μM, simulating clinical use cases such as fluorescence-guided tumor resection. As shown in Fig. 2(l), the system detected clear attenuation signals even at 1  μM, with spectral features becoming progressively more pronounced at higher concentrations. For reference, the standard clinical formulation used in glioma surgery typically involves the intravenous injection of 100 mg SF in 1 mL, corresponding to an initial concentration of ∼265  mM prior to systemic dilution. However, following vascular distribution and tissue uptake, particularly in poorly perfused regions or infiltrative tumor margins, local concentrations can drop significantly. Thus, the ability to detect signals at 1  μM is highly relevant, ensuring that even regions with reduced dye accumulation can still be visualized and delineated with HP1.1, offering crucial guidance during surgery.

### 3.2 Exogenous and Endogenous Chromophore Mapping

To further evaluate HP1.1’s ability to detect diagnostically relevant features in both controlled and biological settings, we designed a series of experiments aimed at mapping exogenous and endogenous chromophores in phantom models and in freshly excised glioma tissue. The overarching aim was to demonstrate the system’s capacity to generate meaningful chromophore maps that can reveal physiological and metabolic features otherwise invisible to the human eye, offering valuable guidance during neurosurgical procedures through near real-time feedback.

**Fig. 3:**
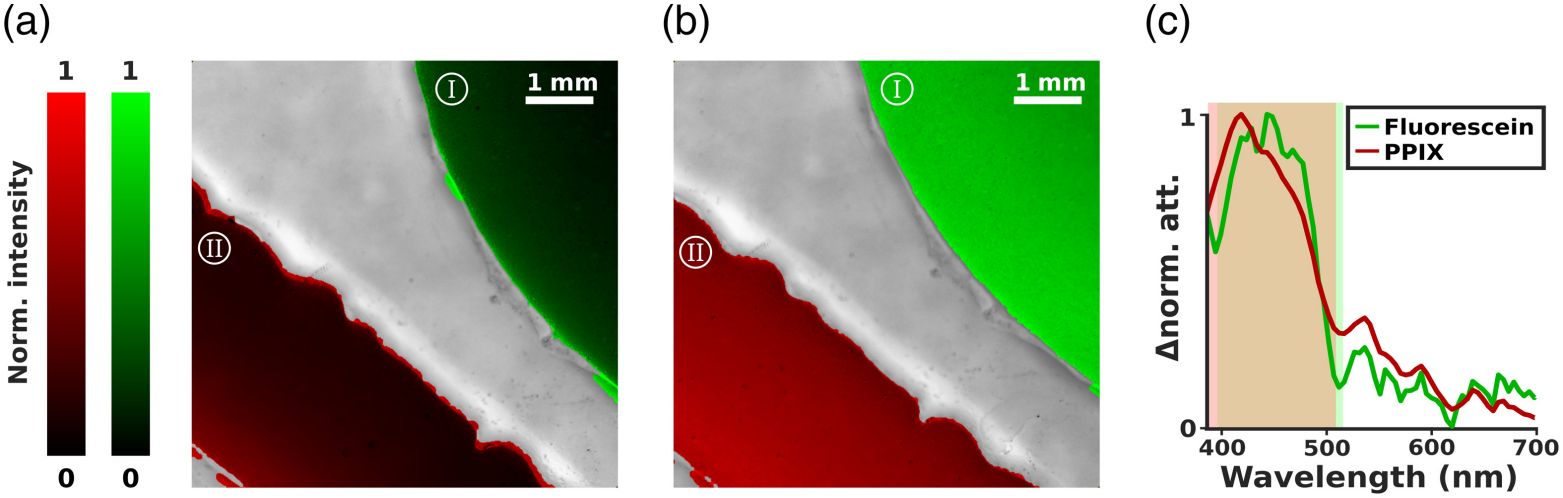
(a), (b) Plastic compartment containing intralipid solutions with fluorescein (upper right) and protoporphyrin IX (lower left) at different concentrations. Colors represent the normalized fluorescein (green) and protoporphyrin (red) concentrations, retrieved from the spectral analysis; colorbars are dimensionless. (c) Difference normalized attenuation spectra from two ROIs in the two compartments; shaded areas represent the range of integration.

Due to the short lifetime and rapid photodegradation of exogenous contrast agents such as fluorescein and protoporphyrin IX (PpIX), it is generally not possible to observe their spectral signatures in glioma tissue *ex vivo*, once the surgical specimen has reached the laboratory. For this reason, we reproduced a simplified, yet physiologically relevant, scenario using liquid phantoms in which the spectral and spatial behavior of these fluorophores could be precisely controlled and assessed. Therefore, in the first experimental setup, a custom 3D-printed plastic compartment featuring two irregularly shaped channels, mimicking blood vessels or other biological structures, was used to compare in the same FOV, two different liquid phantoms. These compartments were filled with the intralipid-fluorescein and intralipid-protoporphyrin solutions described in Materials and Methods, with different concentrations. Figures 3(a) and 3(b) show the plastic compartments filled with the fluorescein solution and PPIX solution in the upper right and lower left corner, respectively. The colors are indicative of the different fluorescein and protoporphyrin IX concentration: in Fig. 3(a), lower concentrations of both fluorophores resulted in visibly attenuated color intensities, whereas Fig. 3(b) displays the same geometry filled with more concentrated solutions, leading to a more intense green and red signal corresponding to fluorescein and PpIX, respectively. Chromophore maps were generated by subtracting a reference spectrum of pure intralipid from each pixel in the hyperspectral data cube, isolating the spectral contribution of the fluorophores. The resulting spectral differences were then integrated over the main absorption bands of fluorescein and PpIX, and the integrals mapped to color intensities: green for fluorescein and red for PpIX. These specific spectral integration windows are shown in Fig. 3(c) as semi-overlapping green and red rectangles. The curves overlaid by these rectangles do not represent raw attenuation spectra, but rather normalized spectral differences computed after the subtraction of the intralipid background, thus emphasizing the selective contribution of each chromophore. The central diagonal section of the phantom, corresponding to the solid plastic wall separating the two channels, could not be filled with liquid, and so, it was excluded from the chromophore quantification using a threshold-based mask, and displayed in its original grayscale in Figs. 3(a) and 3(b). Overall, this liquid phantom-based experiment demonstrated HP1.1’s ability to spectrally resolve multiple exogenous chromophores within the same field of view, even at low concentrations, and to generate contrast-rich maps with high spatial fidelity. This capability is crucial for intraoperative guidance, where visual discrimination of functionally relevant structures may depend on subtle chemical or metabolic differences invisible to the surgeon’s eye.

**Fig. 4:**
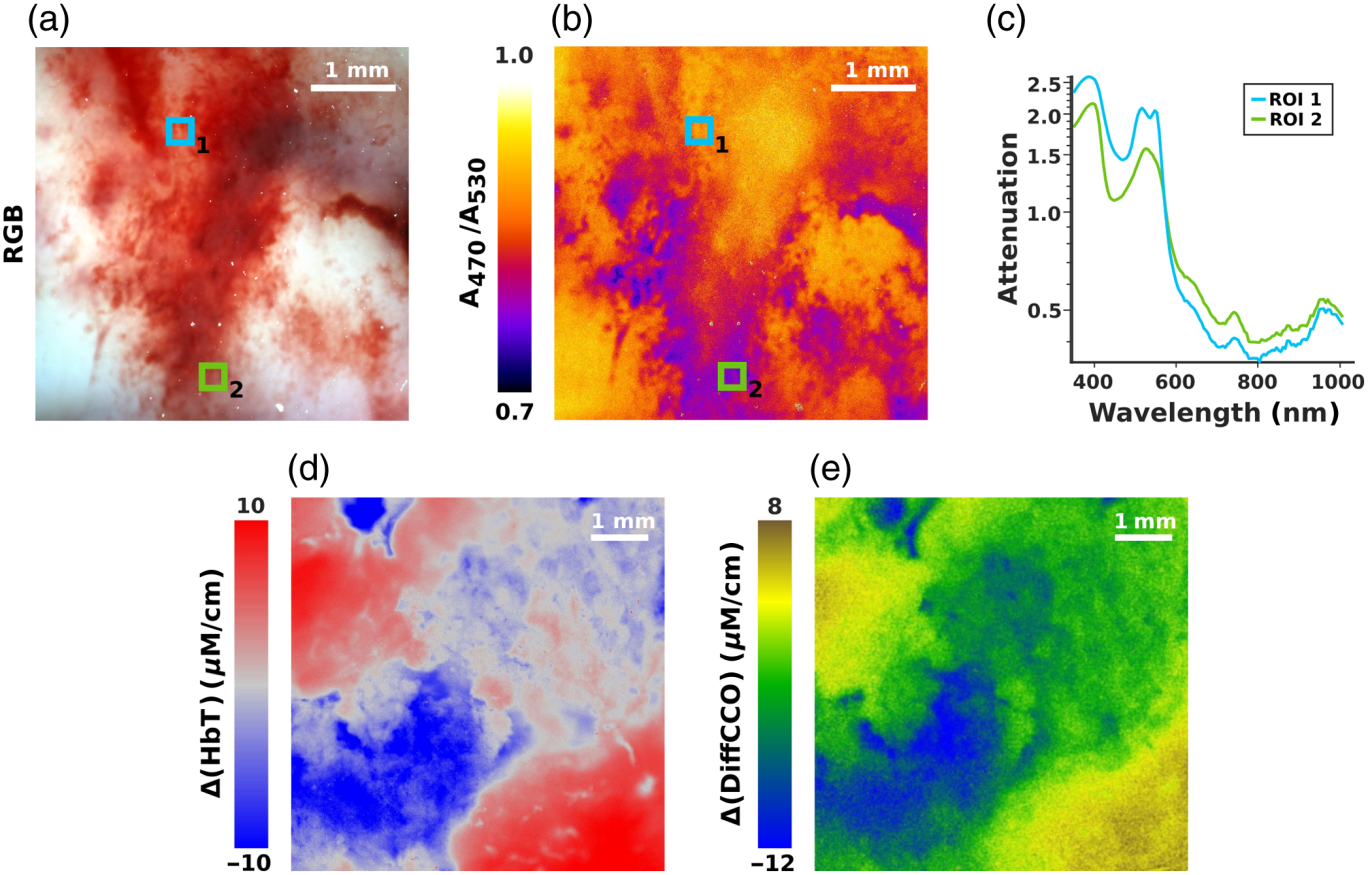
(a) RGB reconstruction of a high-grade glioma showing heterogeneous vascularization. (b) Hemoglobin saturation ($A_{470}/A_{530}$) map of the sample shown in panel (a). (c) Attenuation spectra from the two ROIs shown in panels (a) and (b). (d), (e) Spatial variations inferred maps of total hemoglobin (Δ[HbT]) and differential cytochrome c oxidase difference (Δ[DiffCCO]) relative to the image mean.

Moving from controlled phantoms to biological tissue, we then applied HP1.1 to a freshly resected human glioblastoma sample to explore its ability to spatially map endogenous chromophores, particularly those related to vascular oxygenation and metabolic activity. As a first step, we generated a pseudo-RGB image [Fig. 4(a)] by properly combining the acquisitions at 470, 520, and 670 nm. Although this representation does not convey specific physiological information, it offers a richer and more nuanced visualization compared to conventional grayscale reflectance imaging. Indeed, this image version highlights subtle differences in tissue optical properties that would otherwise be invisible in monochromatic imaging. More importantly, this image served as a reference for defining regions of interest (ROIs) and for direct comparison with the subsequent functional maps, such as hemoglobin oxygenation. In particular, we leveraged the spectral signatures of oxyhemoglobin (${\mathrm{HbO}}_{2}$) and deoxyhemoglobin (Hb), which exhibit well-defined absorption differences in the visible range covered by HP1.1. We computed a pixel-wise oxygenation map by taking the ratio between absorption at 470 nm ($A_{470}$), where oxyhemoglobin absorption dominates, and at 530 nm ($A_{530}$), the isosbestic point, taken as a stable reference unaffected by shifts in oxygenation. This ratiometric approach provides a qualitative but very informative indication of hemoglobin saturation across the FOV. The resulting map shown in Fig. 4(b) reveals spatially heterogeneous oxygenation across the tumor sample, highlighting zones of potential metabolic stress and low perfusion—features of high clinical relevance in glioma surgery. For direct comparison, two regions of interest (ROI 1 and ROI 2) were selected from the RGB image. ROI 1 was located in a well-perfused, presumably oxygenated area, whereas ROI 2 was chosen in a visibly darker zone suspected to be hypoxic. The corresponding spectra shown in Fig. 4(c) confirm these assumptions, displaying spectral features indicative of high ${\mathrm{HbO}}_{2}$ content within ROI 1, and a predominance of deoxygenated hemoglobin in ROI 2. Notably, HP1.1 was applied to resected tumor specimens imaged *ex vivo*, where the vascular circulation is interrupted, and oxygenation levels may no longer reflect the exact *in vivo* conditions. Furthermore, the hyperspectral data primarily represent the superficial tissue layers, which are generally better perfused and more accessible to ambient oxygen compared with deeper, potentially necrotic tumor zones. This could explain the relatively high presence of HbO_2_ signatures observed in our maps.

Moving beyond oxygenation mapping alone, HP1.1 also enabled the extraction of functional information on vascular supply and mitochondrial activity, two parameters that provide further insight into the tumor’s metabolic heterogeneity. These maps were generated pixel-wise by applying a spectral unmixing algorithm based on the Modified Beer–Lambert Law (MBLL) (see Materials and Methods). Instead of providing absolute concentrations, this analysis highlights local deviations from the mean value across the entire image, allowing us to visualize relative spatial fluctuations in vascular density and mitochondrial oxidative metabolism. Specifically, Figs. 4(d) and 4(e) display spatially resolved maps of concentration changes in total hemoglobin ($\Delta [\mathrm{HbT}]=\Delta [{\mathrm{HbO}}_{2}+\mathrm{Hb}]$) and differential cytochrome c oxidase (Δ[DiffCCO]=Δ[oxCCO−redCCO]), respectively, computed from a grade 4 glioma sample. The Δ[HbT] distribution in Fig. 4(d) reveals marked heterogeneity in hemoglobin content, suggesting a non-uniform vascular architecture—a well-documented feature of high-grade gliomas. In parallel, the Δ[DiffCCO] map in Fig. 4(e) shows distinct spatial variations in the redox balance of cytochrome c oxidase, pointing to differences in mitochondrial activity within the tumor area. This kind of metabolic contrast could be relevant for distinguishing regions of viable tissue from more compromised or necrotic areas.

By enabling the visualization of such functional gradients within the tumor microenvironment, HP1.1 proves capable of providing more than just morphological or chemical contrast. The integration of vascular and metabolic readouts into the imaging workflow could support a larger understanding of tumor composition, particularly in settings where rapid, label-free intraoperative insights are required.

### 3.3 Gliomas Characterization and Grading

**Fig. 5:**
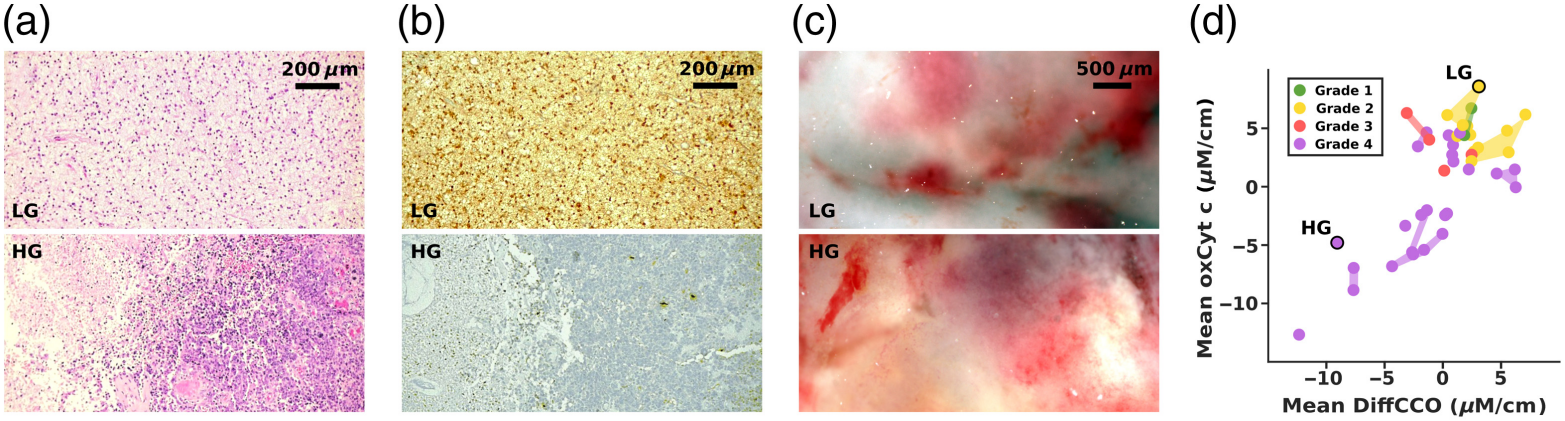
(a–c) Images obtained with H&E staining (a), immunohistochemistry for IDH1 R132H (b), and HP1.1 (c) in RGB colors, of an astrocytoma IDH-mutant, CNS WHO grade 2 (upper sections—LG), and a glioblastoma IDH-wildtype, CNS WHO grade 4 (lower sections—HG), respectively. (d) Mean concentration of oxidized cytochrome c (oxCyt_c) as a function of the mean concentration of differential cytochrome c oxidase (DiffCCO) from glioma biopsies. Each point is color-coded by the sample’s WHO histological grade. Different points originating from the same specimen and acquired from non-overlapping FOVs stand within the same color, shape, or connecting line. The two highlighted dots represent the two samples shown in panels (a–c) (LG and HG).

To assess HP1.1’s potential in supporting glioma grading through metabolic imaging, we compared hyperspectral data acquired from fresh biopsy specimens with the corresponding histopathological classifications derived from adjacent tissue portions. This comparison does not rely on spatial co-registration, as standard histopathological processing profoundly alters the optical and biochemical composition of the tissue, removing lipids, denaturing proteins, and modifying the oxidation states of hemoglobin and cytochromes. Consequently, a pixel-wise correspondence between hyperspectral and histological images would not be physiologically meaningful for the molecular parameters targeted by HP1.1. Instead, the analysis focuses on correlating the metabolic signatures of fresh tissue with the categorical histopathological diagnosis, thus establishing a complementary link between the two approaches. Figures 5(a) and 5(b) show hematoxylin and eosin (H&E) staining and IDH1-R132H immunohistochemistry, respectively, of two representative glioma samples: an IDH-mutant WHO grade 2 astrocytoma (upper panel, low grade [LG]) and an IDH-wildtype WHO grade 4 glioblastoma (lower panel, high grade [HG]). The H&E staining highlights the distinct histological architecture between the two grades: moderate cellularity and mild nuclear atypia in the LG sample, with no necrosis or microvascular proliferation, versus the highly disorganized and necrotic profile of the HG tumor. The immunohistochemical staining further confirms the molecular diagnosis: strong, diffuse cytoplasmic, and nuclear positivity for IDH1 R132H in the LG glioma and almost complete absence of staining in the HG sample, consistent with its wild-type status. Despite this clear distinction at the histological level, these features are not macroscopically evident on the fresh samples. As shown in Fig. 5(c), the RGB reconstructions from the HSI measurements of the two samples reveal no clear visual differences between LG and HG tissues, reflecting the typical intraoperative challenge of distinguishing tumor grade based solely on surface appearance. Nevertheless, to extract physiologically relevant information, we applied spectral unmixing to derive pixel-wise quantitative information about metabolic biomarkers within the brain tumor tissue. In particular, we focused on cytochrome c oxidase (CCO), the terminal enzyme of the mitochondrial electron transport chain that serves as a key indicator of cellular respiration. We used both its oxidized form (oxCCO) and its redox differential (DiffCCO = oxCCO − redCCO) as proxies for oxidative phosphorylation activity. The results are summarized in Fig. 5(d), where for each point analyzed, the mean concentration of oxidized cytochrome c (oxCyt_c) is plotted as a function of DiffCCO. Each data point represents the signal extracted from a single FOV and is color-coded according to WHO grade: Grade 1 (green), 2 (yellow), 3 (red), and 4 (violet). Different points originating from the same specimen and acquired from non-overlapping FOVs stand within the same color shape (or connecting line), indicating that their molecular abundance values cluster within a narrow range. This confirms the internal coherence of the measurements within each sample. The slight dispersion observed among FOVs likely reflects genuine intratumoral heterogeneity in oxygenation and lipid metabolism rather than inconsistencies in histopathological grading, which provides a single label for the entire biopsy.

A distinct stratification of tumor grades based on their metabolic profile is evident. Low-grade gliomas (LGG; grades 1 and 2) predominantly cluster in the upper-right region of the plot. These samples are characterized by elevated concentrations of both oxCCO and DiffCCO, suggesting more active oxidative metabolism and a preserved mitochondrial function. This metabolic signature suggests a viable and well-vascularized tumor microenvironment that supports high aerobic metabolic activity. High-grade gliomas (HGG; grades 3 and 4) occupy a region of the scatter plot located below and to the left of the low-grade gliomas (LGG) cluster, indicating lower concentrations of both oxCCO and DiffCCO. This pattern aligns with the highly aggressive phenotype of HGG, which is typically associated with substantial metabolic stress, hypoxia, and necrosis, resulting in suppressed oxidative metabolism. However, some overlap between classes can be observed, as a few HGG points lie closer to the LGG region, whereas certain LGG points appear more scattered toward the HGG area. This distribution suggests that molecular and metabolic variability exists even within the same histopathological grade and that tumors of different grades may partially share similar biochemical signatures.

Such findings underline the potential of HP1.1 to complement standard histopathology by providing a quantitative view of intra- and inter-class heterogeneity. This metabolic fingerprint, invisible to the naked eye, reveals biologically meaningful differences in tumor physiology and could support real-time intraoperative assessment of tumor aggressiveness.

### 3.4 NADH and FAD Autofluorescence Analysis

Besides the spectral analysis described above, we integrated an autofluorescence acquisition module into our imaging platform to provide additional metabolic contrast based on the intrinsic fluorescence of key cellular cofactors. Specifically, the system leverages the distinct emission properties of NADH and FAD—two endogenous fluorophores involved in central metabolic pathways such as glycolysis and oxidative phosphorylation—to extract metabolic signatures from tissue in a label-free manner. This added dimension of contrast enhances the comprehensiveness of our platform and provides an alternative assessment of glioma heterogeneity and grading.

**Fig. 6:**
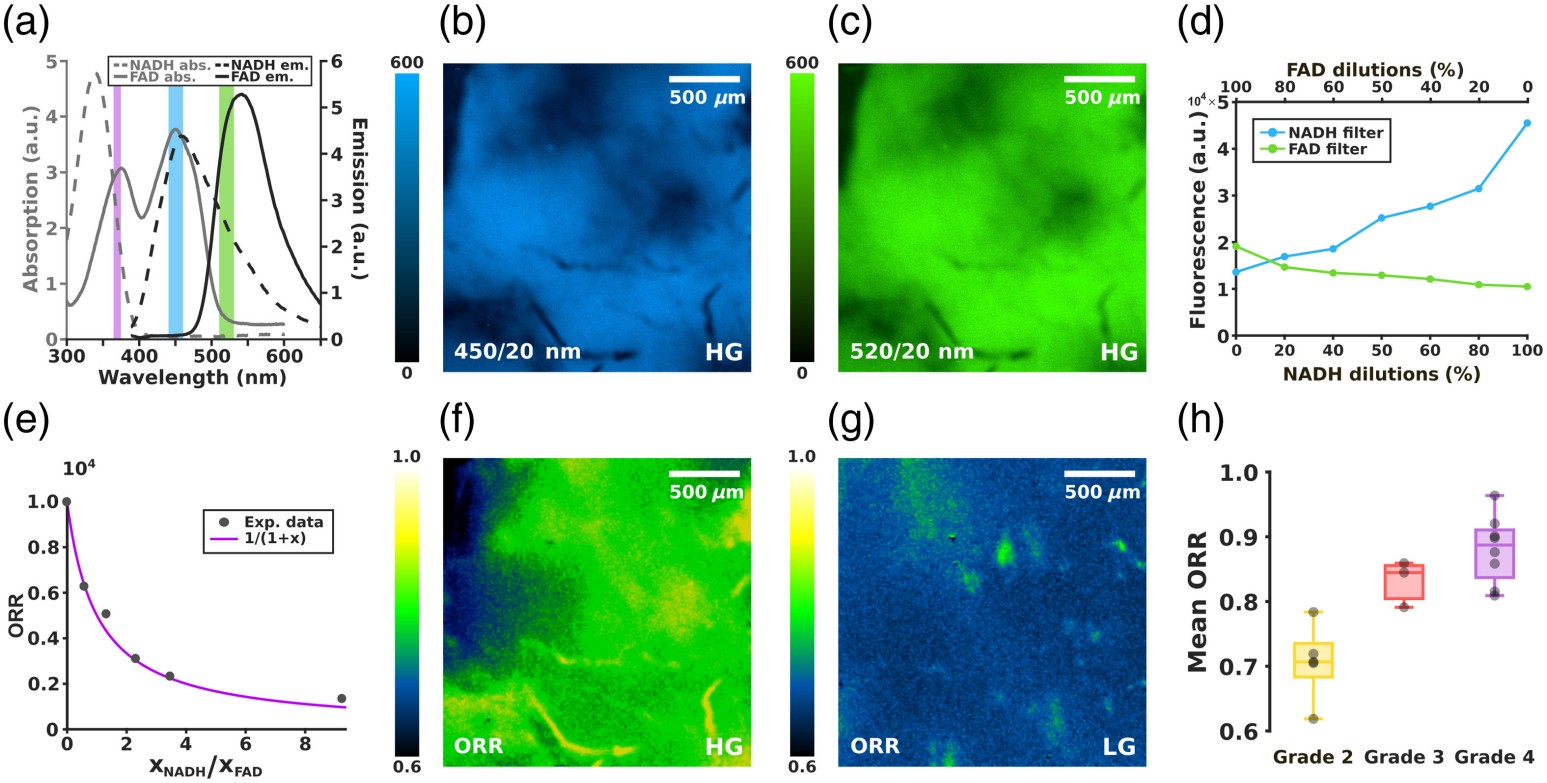
(a) Absorption and emission spectra of NADH and FAD. The purple rectangle represents the LED excitation wavelength (370 nm), whereas the blue and green shaded areas represent the spectral windows of the two filters: 450/20  nm (NADH filter) and 520/20  nm (FAD filter), respectively. (b–c) NADH and FAD autofluorescence images of a high-grade glioma, illuminated with 370 nm light and acquired using 450/20  nm (b) and 520/20  nm (c), acquisition filters, respectively. (d) Mean fluorescence intensities of varying NADH and FAD concentration solutions imaged through the NADH filter (blue line in panel (a) and the FAD filter (green line in panel (a) and calculated over a central region of 1000×1000  pixels. (e) Optical redox ratio (ORR) obtained from solutions with controlled NADH and FAD concentrations. The experimental data, corrected for inter-channel cross-talk, are plotted as a function of the known concentration ratio $x_{\mathrm{NADH}}/x_{\mathrm{FAD}}$. The magenta line represents the expected hyperbolic trend. (f) ORR map of the high-grade glioma sample shown in panels (b) and (c). (g) ORR map of a low-grade glioma sample. (h) Samples’ mean ORR distribution grouped by tumor malignancy grade. Box plots display the median (central line), the 25th and 75th percentiles (box edges), and the whiskers extend to the most extreme data points within 1.5 times the interquartile range ($Q_{75}-Q_{25}$). Dots represent individual samples.

As shown in Fig. 6(a), the absorption and emission spectra of NADH and FAD confirm their distinct optical fingerprints. Although NADH absorbs optimally in the UV range below 360 nm and FAD shows a peak absorption around 370 to 400 nm, we selected a 370-nm LED as the excitation source to achieve a practical and effective compromise. Conversely, the emission bands well match the spectral windows defined by the two filters used in our system, centered at 450±10  nm for NADH and 520±10  nm for FAD, highlighted in blue and green, respectively, in Fig. 6(a). Figures 6(b) and 6(c) show autofluorescence images of a representative high-grade glioma sample acquired through the 450 and 520 nm filters, respectively. Despite using relatively long exposure times (200 ms), the autofluorescence signals in both channels remain weak, relative to the camera’s maximum detectable intensity of 65535 a.u., reflecting the inherently low emission of endogenous fluorophores such as NADH and FAD. Moreover, the two fluorescence images show only minor differences in signal intensity and spatial distribution, suggesting that although the system can distinguish between NADH and FAD, the variations across the tissue surface are relatively small. Therefore, to quantify differences across samples in a consistent manner, we use the optical redox ratio (ORR) as the relevant physical quantity for NADH and FAD autofluorescence analysis:^35^

$$\mathrm{ORR}=\frac{x_{\mathrm{FAD}}}{x_{\mathrm{FAD}}+x_{\mathrm{NADH}}}=\frac{1}{1+x_{\mathrm{NADH}}/x_{\mathrm{FAD}}}$$ (4)

where $x_{\mathrm{FAD}}$ and $x_{\mathrm{NADH}}$ represent the molecular concentrations of FAD and NADH in the sample, which are proportional to the fluorescence intensities emitted by the respective molecular populations. However, it is important to note that these concentrations cannot be directly inferred from the signals measured through the 450 and 520 nm emission filters—that are indicated with $I_{450}$ and $I_{520}$, respectively—as part of each fluorophore’s emission may be detected in the wrong channel due to spectral overlap. To assess the extent of this spectral cross-talk between the NADH and FAD detection channels, we performed calibration measurements using mixed solutions containing both the fluorophores. In each solution, the total liquid volume was kept constant, whereas the relative concentrations of NADH and FAD varied inversely: NADH concentration increased progressively as FAD concentration decreased and vice versa. For each mixture, $I_{450}$ and $I_{520}$ were acquired, computing the mean fluorescence intensities over a central region [Fig. 6(d)]. Notably, also in the boundary cases corresponding to solutions of solely FAD or NADH, a non-zero signal is still detected in both channels. This confirms the presence of spectral cross-talk, consistently with the partial overlap of the emission spectra measured via spectrofluorimetry [Fig. 6(a)]. Therefore, to comprehensively describe the measured intensities $I_{450}$ and $I_{520}$ as a linear combination of the molecular concentration of the two molecules, we use the following matrix form:

$$(\begin{array}{c} I_{450}\\ I_{520} \end{array})=C(\begin{array}{c} x_{N}\\ x_{F} \end{array})=(\begin{array}{cc} a & b\\ c & d \end{array})(\begin{array}{c} x_{N}\\ x_{F} \end{array}),$$ (5)

where the matrix C coefficients take into consideration the different molecular contributions through the filters. The coefficients can be retrieved from calibration measurements at the boundary conditions, where the phantom solutions present solely NADH or FAD:

a=I450NxN^,b=I450FxF^,c=I520NxN^,d=I520FxF^ (6)

where $I_{450}^{N}$ and $I_{520}^{N}$ are the mean fluorescence intensities imaged through 450 and 520 nm filters, respectively, obtained with 100% of NADH, whereas analogously, $I_{450}^{F}$ and $I_{520}^{F}$ are the mean fluorescence intensities with 100% of the FAD sample. In Eq. (6), xN^=281.9  μM and xF^=120.5  μM are the molar concentration used for these boundary cases in the exam. Finally, by inverting Eq. (5) and substituting the coefficients in C, we can retrieve $x_{N}$ and $x_{F}$ for each sample from the measured intensities $I_{450}$ and $I_{520}$. The ORR was therefore computed from Eq. (4) for each calibration solution, showing the expected hyperbolic trend [Eq. (4)], and confirming the validity of the correction method [Fig. 6(e)].

By applying the same ORR calculation at the pixel level to glioma tissue images, we generated ORR maps of a high- and a low-grade glioma, respectively, in Figs. 6(f) and 6(g). These maps offer a spatially resolved view of metabolic activity across the tumor surface, providing at the same time a visual and quantitative readout of the relative abundance of the two fluorophores. Aggregating the ORR values across the entire FOV for multiple samples, we compared the mean ORR across tumors of different malignancy grades. As depicted in the box plot in Fig. 6(h), there is a statistically significant upward trend in mean ORR values with increasing tumor grade. This suggests a metabolic shift associated with glioma progression, potentially reflecting reduced glycolytic activity and increased reliance on oxidative metabolism in higher-grade tumors. This is consistent with the fact that glycolysis leads to NADH production, whereas FAD fluorescence is associated with oxidative phosphorylation.

## 4 Discussion and Conclusion

This study demonstrates the potential of HP1.1 as a comprehensive, rapid, and label-free HSI platform for the characterization of fresh *ex vivo* brain tumor resections. Within this proof-of-concept framework, the system was validated on both solid and liquid phantoms, confirming its optical performance, reliability, temporal stability, and reproducibility. When applied to glioma biopsies of different grades, HP1.1 enabled the extraction of molecular information from key endogenous chromophores—mainly hemoglobin and cytochromes—through tailored spectral unmixing algorithms. Although the dataset remains limited and slightly unbalanced—comprising six low-grade and 22 high-grade gliomas—our findings indicate that this approach is sensitive to physiologically meaningful variations associated with tumor aggressiveness. The overall distribution of molecular abundance values follows a coherent trend consistent with histological grading, reflecting the progressive metabolic shift from low- to high-grade gliomas. Despite the modest number of biological replicates, we deliberately refrained from inferential statistics or classifier development, focusing instead on qualitative and internally consistent trends across multiple FOVs. The reproducibility of these patterns within each specimen reinforces the robustness of the observations and underscores the system’s ability to resolve local metabolic heterogeneity.

HP1.1 is also able to generate two-dimensional concentration maps of hemoglobin oxygen saturation, total hemoglobin, and cytochrome c oxidase activity, revealing local variations related to vascularization, perfusion, and metabolic states. This spatially resolved information is highly relevant for neuro-oncological applications, as oxygen availability and metabolic adaptation are key features of glioma biology. Low-oxygen levels (hypoxia) play a well-established role in driving glioma progression, therapy resistance, and poor prognosis.^36^^,^^37^ In particular, glioblastomas often display highly heterogeneous oxygenation profiles, with hypoxic regions associated with immune suppression and increased malignancy.^38^ Thus, the ability of HP1.1 to visualize oxygenation gradients and metabolic activity at submillimetric resolution may provide valuable intraoperative insight into tissue viability and malignancy, complementing the morphological information offered by MRI and histopathology.

Beyond reflectance-derived parameters, HP1.1 also captures endogenous autofluorescence from NADH and FAD, providing complementary information on redox balance and metabolic signatures that seem to distinguish glioma grades. Although interpreting the ORR trend in gliomas remains challenging, since tumor cells may exploit multiple catabolic pathways for energy production, our findings align with previous studies based on more complex fluorescence lifetime imaging modalities.^28^

Looking ahead, further investigations could explore the application of HP1.1 to other tumor types and to dynamic functional monitoring in preclinical *in vivo* models. Such developments could pave the way toward integrating hyperspectral imaging into neurosurgical workflows as a fast, label-free method for metabolic tissue characterization and intraoperative guidance.

## Data Availability

The datasets generated during the current study are available from the corresponding author upon reasonable request.
